# The Worst War of the World

**Published:** 1917-09-01

**Authors:** 


### THE WORST WAR OF THE WORLD.

### IV. The Germans' Barbarities and the World's Press.

IT seems to us that the time has come when the editor of each newspaper published within the territory of the Allied Nations should tersely and with telling force set out the evidence of the facts which demonstrate, and prove beyond dispute, the hideous inhumanity of the German people, their rulers, and allies in this horrible war.

We have personal evidence of a shocking character of the inhuman and devilish methods pursued against hospitals, the medical, nursing, and ambulance staffs, and the patients under treatment. A valued member of our staff, an experienced, highly-qualified, and able man in the prime of life, was called for the second time to leave his practice at home and join the Armies in the field. He was sent to somewhere in France during the last month. Whilst engaged on his medical duties with the wounded a German shell, charged with infernal ingredients, burst in his vicinity, and he became totally blind. After special and devoted treatment his eyes are mercifully found not to be entirely ruined, but so far he is unable to see to write or to read. This is no solitary case, alas! and is merely typical of the continuous dangers which to-day beset all attendants on the wounded, as well as their patients, when their work entails their presence within reach of the fiendish practices and tactics of the enemies of the Allies, and especially when the latter are Germans.

The *Times* war correspondent at Headquarters in France writes in the *Times* of August 23, 1917: "A new development has arisen, which in any other form of war with any other combatants would be incredible, for the Germans are now deliberately selecting our hospitals and clearing stations for attack. I make this charge dispassionately and in all earnestness. It is not that the Germans may accidentally hit a clearing station, but they deliberately select them for attack and leave all else alone. Three nights ago they bombarded three clearing stations in succession. These had been established in their present positions for two years. The Germans must be perfectly aware of their identity, and there are no objects of legitimate military attack in their immediate neighbourhood." In these attacks on three stations the Germans wounded three women nurses, they killed one and wounded three doctors of the Royal Army Medical Corps, and by a curious irony they killed nine German wounded in their beds. Where the German patients were killed there were many German wounded being tended, in addition to British. The latter remained silent and self-controlled, but the noise and panic of the Germans were indescribable. Thus, when one bomb fell, a German who was being tended by a nurse held up his hands in terror and cried piteously, "Kamerad, kamerad!" to the nurse.

In the *Times* of August 24, page 6, and of August 27, 1917, page 6, the *Times* correspondent writes: "On an airman who was brought down at the Mort Homme a photograph was found showing the hospital at Vadelaincourt clearly marked with its Geneva crosses." At that hospital twenty-two people were killed and about sixty wounded, being hospital orderlies and some wounded soldiers. "The German airman set on fire one of the buildings, which lit up the whole place, and after dropping his bombs fired on the hospital and its inmates with his machine gun. He must have seen what he was doing, for he had descended very low and there is practically not a shadow of doubt that the outrage was deliberate and conscious. It should be noted, too, that in one part of the hospital grounds were lodged 180 wounded German prisoners in the care of the very orderlies who were done to death by the German airman. All of them escaped untouched." The *Times* correspondent speaks of the inhuman brutality of which the Germans have been guilty lately in bombarding, with shells, bombs, and machine-gun bullets, several hospitals in the district. "They seem to grow more and more indifferent to the opinion of the rest of the world. Besides the hospital at Vadelaincourt they have attacked others at Belrupt, Monthairon, and Dugny, and forty-three nurses, nursing orderlies, and wounded soldiers have been killed and fifty-five wounded. The hospital at Dugny was twice shelled during July, and again on nine days during this month, August. Trenches have actually had to be dug round the hospital as a shelter for the nurses, three of whom were killed and five wounded by one shell. Once more I repeat, beyond any manner of doubt, these abominable outrages are deliberately and consciously committed."

We have here indisputable evidence and absolute proof of the Germans' inhuman brutality, which places them outside the pale of civilised humanity. Take again the case of the sinking of hospital ships, though they were conspicuously marked with the red cross and painted the recognised colour of such vessels, so that all might know what they were and whom they carried. At night these ships were illuminated, so the proof is absolute that the outrages on the hospital ships were as deliberate as they were inhuman, and contrary to the law of nations and to the usage of all previous wars. The Germans have practised the murderous policy of placing unarmed and defenceless civilians, when under their control, in front of their soldiers when attacking the forces opposed to them. There is the sworn testimony of a pit-hand of sixteen who, with fifty others, was forced to make trenches for the Germans: "Firing was audible as the French were attacking the main bridge at L——. The Germans put all of us in front of them to act as a shield. The officers drove us to the front with horsewhips, and the soldiers with their rifle-butts. They all hid

☛ Articles I., II., and III. appeared July 28 and August 11 and 18.

**THE WORST WAR OF THE WORLD**—(*continued*).

behind us. We were kept there all the afternoon, with the Germans behind us, firing over our shoulders. . . . We told one another . . . Let the French kill us rather than see the Germans win. . . . Of our party of fifty, forty had been killed."

The *Daily Mail*, under the head "The Holy War," in a published manifesto on the subject, writes: "Such is the treatment of our Allies. What of the treatment of our own people—our captured soldiers, starved, insulted, left naked in winter, tied up by their hands to a post, flogged with rubber whips, and left to die by the hundred of typhus in insanitary camps?"

Then, again, what has characterised the policy of organised evil pursued by the Germans in their colonies? A German statesman, General von Lieberg, has declared: "It is impossible in Africa to get on without cruelty." "In pursuance of this policy they exterminated a whole nation, the Hereros, and through the length and breadth of Africa the natives speak of the German colonies as "the Colonies of the Twenty-five Cuts." In China cruelty was again their policy. The German Emperor told his men to practise it deliberately upon the Chinese. But such terrible cruelties were mere rehearsals of the evil, cruelties, and outrages perpetrated by the Germans in Belgium, in France, in all Europe. Truly has it been declared that the Germans have dragged Christendom back into barbarism. Since the war began, as one writer records, the Germans have raped thousands of women and girls, transported thousands to slavery, and massacred whole streets and villages of people. The evidence of this is ample and complete." Coming to our own doors, he properly admits we British do not need testimony, for the proofs are the mangled bodies of our own innocent women and children, killed in mere wantonness and cruelty by their airmen. To-day's indictment of German inhumanity on land may properly end with the following quotation from the Bishop of Lille's protest, when 25,000 poor French people were deported and forced to work for the enemy. "Thus to dismember the family, by tearing youths and girls from their homes, is not war; it is for us torture, and the worst of tortures—unlimited moral torture. The violation of family rights is doubled by a violation of the sacred demands of morality."

We have no space, nor is it necessary, for us to give a detailed account of the abominable, heartless, inhuman, and barbarous outrages of German submarines on defenceless fishermen, merchantmen, and other seafaring folk, including those on hospital ships. One of the latest unnecessary and deliberately devilish actions of the German sailors and their officers, who man these submarines, is to be found in the testimony of Thomas Bowman, chief engineer of the *Belgian Prince*. Here it is. "The commander ordered the master to step on the submarine. . Then all the crew and officers were ordered aboard, searched, and the lifebelts taken off most of the crew and thrown overboard. . . . The Germans were very abusive towards the crew. After this the German sailors got into the two lifeboats, threw the oars, balers, and gratings overboard, took out the provisions and compasses, and then damaged the lifeboats with an axe. About 9 P.M. the submarine dived and threw everybody in the water without any means of saving themselves. There were only three survivors out of a crew of forty-two men all told."

Who is there in the civilised world who does not remember with horror the fate of the unarmed Cunard liner, the *Lusitania*, struck by two torpedoes and sunk without warning and with a loss of 1,134 lives by a German submarine off the Old Head of Kinsale on May 7, 1915? Language fails to supply adequate words of condemnation and horror at the conduct of a once civilised nation like the Germans, who, by their actions in the Worst War of the World, have set aside the teachings and practice of every religion known to man, and have sunk to a level so deep and awful as to put themselves altogether apart from all other nations in the world, both civilised and uncivilised. It must be manifest to every man and woman everywhere, that, if the human races throughout the world are to continue to live in peace as free people, worshipping God according to their customs and beliefs, and if the world is to continue in wholesome, healthy, ennobling, God-fearing, fair-dealing, humane, and cultured communities of peoples, the Germans and their allies must be beaten in this war, and their militarism and teachings, as exhibited by their conduct and practices, must be broken and annihilated. The will to do the abominations and evils of which we have here given examples has been so welcomed by, and admitted to be in accord with the wishes and will of the German people themselves, that less than this must mean the continuous arming of, and an almost perpetual and continuous warfare between the nations, for all time. What are civilised men and women to think of the small minority of individuals in their midst who express a burning desire to meet the Germans in friendly council and grasp their hands in fellowship? If the declared wish to treat as friends these German overlords, leaders, and perpetrators of these devilish acts, is genuine, the utterers of such sentiments, being residents amongst us, should be placed under restraint for their own protection, and that of their countrymen and countrywomen. If the true inspiration of these expressed wishes of any one or more of them is German gold, they should, on conviction, without a moment's avoidable delay, be hanged by the neck until they are dead.

One word in conclusion. It must be apparent to all true-hearted men and women, everywhere, that the proved facts, we have here set forth, should be continuously kept before the entire people of all the Allied nations. The best way, probably, to secure this end would be that every newspaper, and especially those with the largest circulation and the greatest influence, should adopt the course pursued by the *Daily Mail*, in printing a statement like this, or that headed "The Holy War" from which we have quoted, which appears on the first page of the *Daily Mail* of August 25, 1917. That page

**THE WORST WAR OF THE WORLD**—(*continued*).

concludes with words that are specially applicable to the present position of the Allies, and their united aims, "VICTORY BRINGETH PEACE FINALLY."

Half-measures, an arranged peace without victory, can only result in an aggravation of the horrors already perpetrated by the Germans and their allies, and the indefinite continuance of similar outrages attempted and often sucessfully perpetrated, both upon kindred, and especially upon weaker nations, throughout the world. Let each reader take the facts we have given to heart, store them in the memory, and circulate them wherever possible. This is every free man and woman's duty. Its fulfilment will be a portion of their individual contribution to the ultimate securing of the victory, which alone can bring peace, ultimately, to the nations.

